# Low‐level viraemia as a risk factor for virologic failure in children and adolescents living with HIV on antiretroviral therapy in Tanzania: a multicentre, retrospective cohort study

**DOI:** 10.1002/jia2.26474

**Published:** 2025-05-12

**Authors:** Kevin P. McKenzie, Duc T. Nguyen, Lilian B. Komba, Eunice W. Ketang'enyi, Neema E. Kipiki, Evance N. Mgeyi, Lumumba F. Mwita

**Affiliations:** ^1^ Department of Pediatrics Baylor College of Medicine Houston Texas USA; ^2^ Texas Children's Global Health Network Houston Texas USA; ^3^ Baylor College of Medicine Children's Foundation – Tanzania Mbeya Tanzania; ^4^ Baylor College of Medicine Children's Foundation – Tanzania Mwanza Tanzania

**Keywords:** HIV, paediatric, viraemia, virologic failure, sub‐Saharan Africa, treatment failure

## Abstract

**Introduction:**

Viral load (VL) of 1000 copies/ml or greater is commonly used to define virologic failure (VF) in children and adolescents living with HIV (CALHIV) in low‐ and middle‐income countries (LMICs). However, evidence in adults suggests that low‐level viraemia (LLV) (VL 50–999 copies/ml) increases the risk of subsequent VF. There is limited research on LLV in CALHIV.

**Methods:**

This study retrospectively reviewed VL data from Baylor College of Medicine Children's Foundation—Tanzania (sites in Mbeya and Mwanza) collected between January 2015 and December 2022. CALHIV (0−19 years) on antiretroviral therapy for ≥6 months with at least one VL <50 copies/ml plus ≥2 subsequent VLs were included. VF was defined as both VL ≥1000 and ≥200 copies/ml. Multivariable Cox regression models were used to assess the association between LLV and VF, reporting adjusted hazard ratios (aHR) with 95% confidence intervals (CI).

**Results:**

Among 2618 CALHIV included in the outcome analysis (median age 13.2 years, 52.5% female), 81.9% were on first‐line dolutegravir‐based regimens and LLV was found in 40.5%. CALHIV with LLV had an increased risk of VF with aHRs of 1.63 (CI 1.38−1.91) (VL ≥1000 copies/ml) and 3.85 (3.33, 4.46) (VL ≥200 copies/ml). When stratifying by LLV (50−199, 200–399 and 400–999 copies/ml), all levels were associated with increased risk for VF (VL ≥1000 copies/ml) with aHRs of 1.39 (1.13, 1.69), 1.69 (1.33, 2.16) and 2.03 (1.63, 2.53). When VF was defined as VL ≥200 copies/ml, the corresponding aHRs were 1.41 (1.15, 1.72), 7.99 (6.68, 9.57) and 9.37 (7.85, 11.18).

**Conclusions:**

LLV is associated with a greater risk of VF in CALHIV. The risk of VF increases with higher levels of LLV. This study provides further evidence for revising guidelines in LMICs that define VF as VL ≥1000 copies/ml.

## 1 INTRODUCTION

Despite the introduction of new and better tolerated antiretroviral therapy (ART) formulations for children and adolescents living with HIV (CALHIV), CALHIV remain at high risk for delay in treatment [1], virologic failure (VF) [2, 3], viraemia [4, 5] and ART drug resistance [6, 7]. In 2022, according to UNAIDS, there were an estimated 1.5 million CALHIV (ages 0–14) including 130,000 new acquisitions, with approximately 45% originating in eastern and southern Africa [1]. Among all CALHIV, only 57% had access to ART [1], and of those on ART, only around 60–75% had achieved virologic suppression [2]. This percentage is far below the Joint United Nations Programme on HIV/AIDS target of 90% virologic suppression for 2020 [8], let alone the more ambitious goal of 95% for 2030 [9].

The issue of virologic suppression is further complicated by a lack of consensus on the definition of VF. While many high‐income countries use a viral load (VL) of <200 copies/ml (e.g. USA [10]) or <50 copies/ml (e.g. the EU [11]) to define VF, the majority of low‐ and middle‐income counties (LMICs) follow the WHO guidelines which recommend a VL cutoff of 1000 copies/ml [12]. Unfortunately, these variations in VF definition are largely historical and do not reflect current laboratory capabilities in most countries including LMICs. Moreover, there are comparatively few studies examining VF in CALHIV, casting doubt on whether current guidelines concerning VF truly apply.

Low‐level viraemia (LLV) is defined as a VL that falls between undetectable (which may vary depending upon equipment used but is usually <50 copies/ml) and 1000 copies/ml. It is a concept that has gained increasing attention in recent years due to the widespread adoption of routine VL monitoring worldwide coupled with unclear or absent attention to LLV in most guidelines. As a result, a growing number of CALHIV present to care with LLV but are rarely provided with additional services since, although they are not virologically suppressed, they do not meet the criteria for VF according to local definitions.

Mounting evidence points to LLV as an independent risk factor for VF [13, 14, 15, 16, 17], but few studies concerning LLV have been conducted in children. Moreover, LLV has also been shown to lead to the development of ART resistance [18, 19, 20] and poor health outcomes [21], including all‐cause and AIDS‐related mortality [22]. More focus has been placed on LLV due to the growing concern in LMICs about the development of and dissemination of drug resistance mutations (DRMs), especially in the case of dolutegravir (DTG) [23].

The CLOVES (CALHIV on ART with viraemia in sub‐Saharan Africa) project aims to investigate the underlying causes of viraemia and VF in young people in sub‐Saharan Africa (SSA) and how to address them. The project's primary objective is to analyse clinically focused indicators to help clinicians serving CALHIV make decisions on how to prevent and optimize the management of VF.

## 2 METHODS

### 2.1 Participants

Baylor College of Medicine Children's Foundation—Tanzania was established in 2009 and founded two paediatric centres of excellence (COE) in Mwanza, in north‐west Tanzania, and Mbeya, in southern Tanzania. The centres serve as referral hubs for clinics throughout the Lake (LZ) and Southern Highland Zones (SHZ) in Tanzania and commonly enrol late presenting CALHIV or those who have failed treatment at other facilities. Approximately 7000 CALHIV are currently receiving treatment for HIV, tuberculosis (TB), malnutrition and other services at these two COEs.

**Figure 1:**
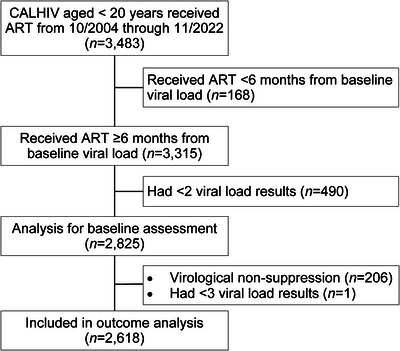
Flowchart of the study population. ART, antiretroviral therapy; CALHIV, children and adolescents living with HIV.

For this analysis, retrospective data was extracted from the electronic medical records, as well as the national database. All CALHIV who had been on ART for at least 6 months and had at least two VLs taken were included (Figure 1). With the purpose of minimizing selection bias in the analysis, CALHIV were included in the outcome analysis only if they achieved a VL of less than 50 copies/ml and then had two subsequent VLs after that time point. To improve the applicability of the results, the analysis was performed with two different VF definitions: ≥1000 and ≥200 copies/ml. LLV was defined as any VL from 50 to 999 copies/ml.

A waiver of consent was provided by the Baylor College of Medicine Institutional Review Board for the analysis of observational data in this cohort.

### 2.2 Local environment

ART was introduced in Tanzania in 2004 and was followed by a rapid scale‐up [24]. Most CALHIV were placed on fixed dose combinations (FDCs) of stavudine (d4T), lamivudine (3TC) and nevirapine (NVP) and later, zidovudine (AZT), 3TC and NVP. Protease inhibitor (PI) therapy replaced non‐nucleoside reverse transcriptase inhibitors (NNRTI) in 2018 with most CALHIV started on an ABC‐3TC FDC and lopinavir/ritonavir (LPV/r), either in syrup or dissolving granule form. Unfortunately, these LPV/r formulations proved to be unpalatable to many children [25] and contributed to poor VL outcomes, especially in those under 5 years of age. DTG was introduced in 2019, initially as a 50‐milligram tablet. The 10‐milligram dispersible tablet became available in 2022, with paediatric formulations of LPV/r rapidly phased out.

VL testing was introduced in Tanzania in 2015 [26] and the first VL was performed at Baylor Foundation Tanzania clinics in that year. Per Tanzanian national guidelines [27], CALHIV are considered virologically suppressed at a VL <1000 copies/ml and only require annual VL testing (all receive two VL tests in their first year on ART). CALHIV with VL greater than 1000 copies/ml are required to undergo 3 monthly sessions of enhanced adherence counselling and a repeat VL is conducted after these sessions are completed. If the VL is still ≥1000 copies/ml and the patient is on their first ART regimen, they should be switched to a different regimen. If they have a history of ART switching due to VF, a genotype should be performed, and an adequate regimen should be selected based on the results. Currently, there are no guidelines concerning the management of LLV in CALHIV.

In 2022, a Viremia Clinic was started in Mbeya to address the problem of VF and emphasized predicting and preventing its occurrence.

### 2.3 Statistical analysis

Participant characteristics were reported as frequencies and proportions for categorical variables and as median and interquartile range (IQR) for continuous variables. Differences in participant characteristics between categories of HIV VL were determined by the chi‐square or Fisher's exact tests for categorical variables and the Kruskal−Wallis test for continuous variables. Cumulative incidence of VF, defined as a single VL ≥1000 copies/ml, and of first VL ≥200 copies/ml were estimated using the Kaplan−Meier statistic. Differences in the VF incidence between VL groups were compared using the log‐rank test. Multivariable Cox regression modelling was conducted to determine risk factors associated with having VL ≥1000 copies/m and first VL ≥200 copies/ml in participants who had previously attained virological suppression (first VL ≤50 copies/ml) and had at least three VL results. Four separate models were fitted with the same covariates, except for the following differences: model 1 included the categorical ART regimen and dichotomous LLV variables; model 2 included the categorical ART regimen and categorical LLV variables; model 3 included the dichotomous ART regimen and dichotomous LLV variables; and model 4 included the dichotomous ART regimen and categorical LLV variables. The selection of variables for the multivariable models was conducted using the least absolute shrinkage and selection operator method with the cross‐validation selection option [28, 29], and based on clinical importance. Trends of percent VL <1000 copies/ml, <50 copies/ml and LLV were evaluated using a non‐parametric trend test and depicted using a line graph. Analyses were performed using Stata version 18.5 (StataCorp LLC, College Station, TX, USA). A *p*‐value of <0.05 was considered statistically significant.

## 3 RESULTS

A total of 2618 CALHIV were included in the outcome assessment with 7364 person‐years of follow‐up (Table 1). Participants from SHZ represented 55.1% of the total. The median age was 13.2 years (IQR, 9.7−16.7), 73.3% were adolescents (ages 10–19 years) and 52.6% were female. Most participants had a WHO Stage of III or IV (56.1%) and 2.3% had CD4 counts of less than 200 cells/mm^3^. Concurrent TB was found in 15.6% of the participants. ART was started at a median age of 4.1 years (IQR, 1.5−7.9) and the median duration was 7.1 years (IQR, 4.0−9.5). Most participants had baseline ART regimens of ABC and 3TC plus LPV/r (23.5%) or AZT and 3TC plus an NNRTI (44.3%). Current regimens consisted of tenofovir (TDF) and 3TC plus DTG (55.4%) and ABC‐3TC‐DTG (26.5%) with 11.8% on second line. Overall, 83.1% of the participants had DTG in their current regimens. Participants had a median of one VL measurement per year (IQR, 1.0−1.2).

**Table 1:** Patient characteristics, by category of first viral load results—cohort of individuals included in the outcome analysis

	Total	No LLV	LLV	
Variables	(*n* = 2618)	(*n* = 1558)	(*n* = 1060)	*p*‐value
Age (years), median (IQR)	13.2 (9.7, 16.7)	12.8 (9.2, 16.5)	13.6 (10.2, 17.1)	<0.001
Age group (years)				<0.001
<5	137 (5.2)	112 (7.2)	25 (2.4)	
5–9	561 (21.4)	349 (22.4)	212 (20.0)	
10–14	905 (34.6)	523 (33.6)	382 (36.0)	
15–19	1015 (38.8)	574 (36.8)	441 (41.6)	
Age at ART start, median (IQR)	4.1 (1.5, 7.9)	4.0 (1.4, 7.8)	4.3 (1.7, 8.1)	0.071
Sex				0.43
Female	1376 (52.6)	809 (51.9)	567 (53.5)	
Male	1242 (47.4)	749 (48.1)	493 (46.5)	
Base WHO Stage				<0.001
I	657 (25.1)	435 (27.9)	222 (20.9)	
II	492 (18.8)	297 (19.1)	195 (18.4)	
III	663 (25.3)	379 (24.3)	284 (26.8)	
IV	806 (30.8)	447 (28.7)	359 (33.9)	
Current WHO Stage				<0.001
I	191 (7.3)	157 (10.1)	34 (3.2)	
II	214 (8.2)	152 (9.8)	62 (5.8)	
III	687 (26.2)	403 (25.9)	284 (26.8)	
IV	1526 (58.3)	846 (54.3)	680 (64.2)	
Current weight (kg), median (IQR)	33.0 (23.9, 47.0)	32.0 (22.5, 46.0)	35.0 (25.0, 48.0)	<0.001
Recent CD4 results (cells/mm^3^), median (IQR)	952.0 (676.0, 1283.0)	968.5 (686.0, 1321.0)	919.0 (665.0, 1234.0)	0.001
Recent CD4 results (cells/mm^3^)				0.096
≥500	2351 (89.8)	1414 (90.8)	937 (88.4)	
200–499	206 (7.9)	108 (6.9)	98 (9.2)	
<200	61 (2.3)	36 (2.3)	25 (2.4)	
Time from ART start to first VL				0.034
6–12 months	764 (29.2)	472 (30.3)	292 (27.5)	
1–5 years	1335 (51.0)	762 (48.9)	573 (54.1)	
>5 years	519 (19.8)	324 (20.8)	195 (18.4)	
Years from ART start to last VL, median (IQR)	7.1 (4.0, 9.5)	6.6 (3.4, 9.2)	7.6 (5.3, 9.7)	<0.001
Tuberculosis				0.17
No	2209 (84.4)	1302 (83.6)	907 (85.6)	
Yes	409 (15.6)	256 (16.4)	153 (14.4)	
Baseline ART regimen				<0.001
ABC+3TC+LPV/r	614 (23.5)	405 (26.0)	209 (19.8)	
AZT+3TC+NVP	656 (25.1)	369 (23.7)	287 (27.2)	
AZT+3TC+EFV	503 (19.3)	270 (17.4)	233 (22.1)	
ABC+3TC+EFV	286 (10.9)	163 (10.5)	123 (11.6)	
TDF+3TC+DTG	124 (4.7)	90 (5.8)	34 (3.2)	
d4T+3TC+NVP	200 (7.7)	121 (7.8)	79 (7.5)	
Other first line	202 (7.7)	122 (7.8)	80 (7.6)	
Second line	27 (1.0)	16 (1.0)	11 (1.0)	
Current ART regimen				<0.001
TDF+3TC+DTG	1450 (55.4)	819 (52.6)	631 (59.5)	
ABC+3TC+DTG	694 (26.5)	465 (29.8)	229 (21.6)	
Other first line	165 (6.3)	111 (7.1)	54 (5.1)	
Second line	309 (11.8)	163 (10.5)	146 (13.8)	
Current ART regimens				0.01
First line	2309 (88.2)	1395 (89.5)	914 (86.2)	
Second line	309 (11.8)	163 (10.5)	146 (13.8)	
Any DTG in current ART regimens				0.39
No	442 (16.9)	255 (16.4)	187 (17.6)	
Yes	2176 (83.1)	1303 (83.6)	873 (82.4)	
Any DTG in current second‐line ART regimens				0.75
No	2599 (99.3)	1546 (99.2)	1053 (99.3)	
Yes	19 (0.7)	12 (0.8)	7 (0.7)	
Number of VLs per year, median (IQR)^a^	1.0 (1.0, 1.2)	1.0 (1.0, 1.2)	1.2 (1.0, 1.3)	<0.001
Follow‐up time (person‐year)^a^	7364	6051	1312	NA
Site				<0.001
SHZ	1442 (55.1)	657 (42.2)	785 (74.1)	
LZ	1176 (44.9)	901 (57.8)	275 (25.9)	

*Note*: Values are in frequency and % unless otherwise specified. Differences in participant characteristics between groups (LLV vs. no LLV) were determined by the chi‐square or Fisher's exact tests for categorical variables and the Kruskal−Wallis test for continuous variables.

Abbreviations: 3TC, lamivudine; ABC, abacavir; ART, antiretroviral therapy; AZT, zidovudine; d4T, stavudine; DTG, dolutegravir; EFV, efavirenz; IQR, interquartile range; LLV, low‐level viraemia; LPV/r, lopinavir/ritonavir; LZ, Lake Zone, Tanzania; NA, not applicable; NVP, nevirapine; SHZ, Southern Highlands Zone, Tanzania; TDF, tenofovir disoproxil fumarate; VL, viral load; WHO, World Health Organization.

^a^The median (IQR) number of VLs per year and follow‐up time (person‐years) were 1.0 (1.0, 1.3) and 4167 at SHZ, and 1.0 (1.0, 1.2) and 3197 at LZ, respectively.

**Figure 2:**
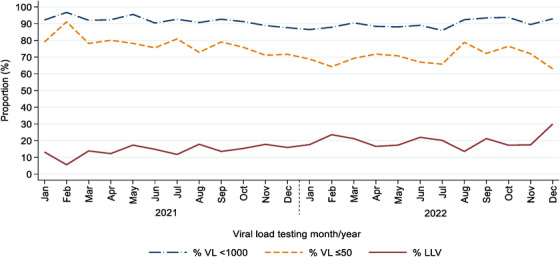
Trends of % viral load (VL) <1000, <50 and low‐level viraemia (LLV), non‐parametric. Non‐parametric trend test: For % VL<1000: *z* = −1.97; *p* = 0.049. For % VL≤50: *z* = −9.8; *p*<0.001. For % LLV: *z* = 10.3; *p*<0.001.

When examining the trends of suppression over time since 2021 (Figure 2), we found that overall virologic suppression (VL <1000 copies/ml) has been decreasing (non‐parametric trend test *z* = −1.97, *p* = 0.049). Additionally, virologic suppression at VL <50 copies/ml has been decreasing (*z* = −9.8, *p* <0.001) with a concurrent rise in LLV (*z* = 10.3, *p* <0.001).

Comparing participants who had experienced LLV after initial suppression versus those who did not (Table 1), LLV participants were older (median age 13.6 vs. 12.8 years, *p* <0.001) with concomitantly lower CD4 (919 vs. 969 cells/mm^3^, *p* = 0.001) and greater time on ART (7.6 vs. 6.6 years, *p* <0.001). LLV participants more commonly had baseline WHO Stages of III or IV (26.8% and 64.2% vs. 25.9% and 54.3%, *p* <0.001). Those without LLV were more often started on a PI (26.0% vs. 19.8%, *p* <0.0001), were more likely to currently be taking ABC‐3TC‐DTG (29.8% vs. 21.6%, *p* <0.001) and be on a first‐line therapy (89.5% vs. 86.22%, *p* = 0.01). There were no significant differences found in the age at ART initiation, sex, number with severe immunosuppression, TB or DTG in the current regimen.

When defining VF as 1000 or greater copies/ml, multivariable Cox regression analysis demonstrated that age <5 years (adjusted hazard ratio [aHR]: 2.20; 95% confidence interval [CI], 1.26−3.81; *p* = 0.01) and immunosuppression (CD4 200–499 cells/mm^3^ [aHR: 1.72; 95% CI, 1.33−2.23; *p*<0.001] or <200 cells/mm^3^ [aHR: 2.60; 95% CI, 1.73−3.89; *p*<0.001]) were associated with VF (Table 2). Being on DTG was protective (aHR: 0.55; 95% CI, 0.45−0.67; *p*<0.001). LLV was associated with VF at all levels studied: overall (aHR: 1.63; 95% CI, 1.38−1.91; *p*<0.001), 51–199 copies/ml (aHR: 1.39; 95% CI, 1.13−1.69, *p* = 0.001), 200–399 copies/ml (aHR: 1.69; 95% CI, 1.33−2.16; *p*<0.001) and 400–999 copies/ml (aHR: 2.03; 95% CI, 1.63−2.53; *p*<0.001) (Table 3 and Table S1).

**Table 2:** Characteristics associated with virologic failure (viral load ≥1000 copies/ml), univariable Cox proportional hazards regression

Variables	No VF	VF	Univariable	
	(*n* = 1992)	(*n* = 626)	HR (95% CI)	*p*‐value
Age (years), median (IQR)	12.8 (9.3, 16.5)	14.4 (10.8, 17.5)	1.02 (1.00, 1.04)	0.02
Age group (years)				
<5	122 (6.1)	15 (2.4)	1.74 (1.01, 3.00)	0.047
5–9	458 (23.0)	103 (16.5)	(reference)	
10–14	682 (34.2)	223 (35.6)	1.01 (0.80, 1.28)	0.93
15–19	730 (36.6)	285 (45.5)	1.28 (1.02, 1.60)	0.03
Age at ART start, median (IQR)	4.0 (1.5, 7.9)	4.8 (1.6, 8.0)	1.03 (1.01, 1.05)	0.01
Sex				
Female	1037 (52.1)	339 (54.2)	(reference)	
Male	955 (47.9)	287 (45.8)	0.98 (0.83, 1.14)	0.77
Base WHO Stage				
I	544 (27.3)	113 (18.1)	(reference)	
II	365 (18.3)	127 (20.3)	1.29 (1.00, 1.66)	0.049
III	485 (24.3)	178 (28.4)	1.27 (1.00, 1.61)	0.047
IV	598 (30.0)	208 (33.2)	1.23 (0.98, 1.54)	0.08
Current WHO Stage				
I	172 (8.6)	19 (3.0)	(reference)	
II	176 (8.8)	38 (6.1)	1.40 (0.81, 2.42)	0.23
III	526 (26.4)	161 (25.7)	1.65 (1.03, 2.66)	0.04
IV	1118 (56.1)	408 (65.2)	1.82 (1.15, 2.89)	0.01
Current weight (kg), median (IQR)	32.3 (22.9, 45.8)	37.1 (26.8, 49.7)	1.00 (1.00, 1.01)	0.16
Recent CD4 results (cells/mm^3^), median (IQR)	982.0 (712.0, 1323.0)	827.5 (585.0, 1165.0)	1.00 (1.00, 1.00)	<0.001
Recent CD4 results (cells/mm^3^)				
≥500	1821 (91.4)	530 (84.7)	(reference)	
200–499	136 (6.8)	70 (11.2)	2.03 (1.58, 2.61)	<0.001
<200	35 (1.8)	26 (4.2)	3.10 (2.09, 4.59)	<0.001
Time from ART start to first VL				
6–12 months	618 (31.0)	146 (23.3)	(reference)	
1–5 years	993 (49.8)	342 (54.6)	0.86 (0.70, 1.04)	0.12
>5 years	381 (19.1)	138 (22.0)	0.91 (0.72, 1.15)	0.41
Time from ART start to last VL, median (IQR)	6.7 (3.6, 9.2)	8.1 (6.0, 10.0)	0.99 (0.97, 1.02)	0.67
Tuberculosis				
No	1679 (84.3)	530 (84.7)	(reference)	
Yes	313 (15.7)	96 (15.3)	1.09 (0.88, 1.36)	0.44
Baseline ART regimens				
ABC+3TC+LPV/r	504 (25.4)	110 (17.6)	(reference)	
AZT+3TC+NV	477 (24.0)	179 (28.6)	0.86 (0.67, 1.09)	0.2
AZT+3TC+EFV	357 (18.0)	146 (23.4)	0.86 (0.67, 1.11)	0.25
ABC+3TC+EFV	215 (10.8)	71 (11.4)	0.89 (0.66, 1.20)	0.45
TDF+3TC+DTG	115 (5.8)	9 (1.4)	0.77 (0.39, 1.52)	0.45
d4T+3TC+NVP	145 (7.3)	55 (8.8)	0.92 (0.67, 1.28)	0.63
ABC+3TC+DTG	157 (7.9)	45 (7.2)	1.06 (0.75, 1.49)	0.76
Other	17 (0.9)	10 (1.6)	1.72 (0.90, 3.29)	0.1
Current ART regimens				
TDF+3TC+DTG	1088 (54.6)	362 (57.8)	(reference)	
ABC+3TC+DTG	578 (29.0)	116 (18.5)	0.91 (0.74, 1.12)	0.38
ABC+3TC+LPV/r	137 (6.9)	28 (4.5)	1.51 (1.03, 2.22)	0.04
Other	189 (9.5)	120 (19.2)	2.23 (1.81, 2.74)	<0.001
Current ART regimens				
First line	1803 (90.5)	506 (80.8)	(reference)	
Second line	189 (9.5)	120 (19.2)	2.23 (1.83, 2.73)	<0.001
Any DTG in current ART regimens				
No	306 (15.4)	136 (21.7)	(reference)	
Yes	1686 (84.6)	490 (78.3)	0.49 (0.40, 0.59)	<0.001
Any DTG in current second‐line ART regimens				
No	1980 (99.4)	619 (98.9)	(reference)	
Yes	12 (0.6)	7 (1.1)	1.87 (0.89, 3.93)	0.10
Low‐level viraemia (LLV)				
No LLV	1301 (65.3)	257 (41.1)	(reference)	
LLV	691 (34.7)	369 (58.9)	1.71 (1.45, 2.00)	<0.001
Viral load (copies/ml)				
≤50	1301 (65.3)	257 (41.1)	(reference)	
51–199	387 (19.4)	155 (24.8)	1.44 (1.18, 1.75)	<0.001
200–399	150 (7.5)	91 (14.5)	1.79 (1.41, 2.27)	<0.001
400–999	154 (7.7)	123 (19.6)	2.14 (1.72, 2.65)	<0.001

*Note*: Values are in frequency and % unless otherwise specified.

Abbreviations: 3TC, lamivudine; ABC, abacavir; ART, antiretroviral therapy; AZT, zidovudine; d4T, stavudine; DTG, dolutegravir; EFV, efavirenz; IQR, interquartile range; LLV, low‐level viraemia; LPV/r, lopinavir/ritonavir; NVP, nevirapine; TDF, tenofovir disoproxil fumarate; VF, virologic failure; VL, viral load; WHO, World Health Organization.

**Table 3:** Characteristics associated with virologic failure (viral load ≥1000 copies/ml), multivariable Cox proportional hazards regression

	Multivariable, model 1^a^		Multivariable, model 2^b^	
Variables	HR (95% CI)	*p*‐value	HR (95% CI)	*p*‐value
Age group (years)				
<5	2.20 (1.26, 3.81)	0.01	2.26 (1.30, 3.92)	0.004
5–9	(reference)		(reference)	
10–14	0.96 (0.73, 1.25)	0.74	0.96 (0.74, 1.26)	0.79
15–19	1.14 (0.85, 1.52)	0.38	1.14 (0.85, 1.51)	0.39
Sex				
Female	(reference)		(reference)	
Male	0.93 (0.79, 1.09)	0.36	0.93 (0.79, 1.09)	0.36
Current WHO Stage				
I	(reference)		(reference)	
II	1.25 (0.72, 2.19)	0.43	1.27 (0.73, 2.21)	0.4
III	1.28 (0.79, 2.08)	0.32	1.26 (0.77, 2.05)	0.36
IV	1.42 (0.89, 2.28)	0.14	1.42 (0.88, 2.27)	0.15
Recent CD4 results (cells/mm^3^)				
≥500	(reference)		(reference)	
200–499	1.72 (1.33, 2.23)	<0.001	1.70 (1.31, 2.20)	<0.001
<200	2.60 (1.73, 3.89)	<0.001	2.59 (1.73, 3.88)	<0.001
Tuberculosis				
No	(reference)		(reference)	
Yes	1.10 (0.89, 1.38)	0.38	1.13 (0.91, 1.42)	0.27
Current ART regimens				
TDF+3TC+DTG	(reference)		(reference)	
ABC+3TC+DTG	1.00 (0.76, 1.31)	1.00	0.99 (0.76, 1.30)	0.97
ABC+3TC+LPV/r	1.54 (1.02, 2.31)	0.04	1.54 (1.03, 2.32)	0.04
Other	2.00 (1.61, 2.47)	<0.001	1.99 (1.61, 2.46)	<0.001
Any DTG in current ART regimens				
No	NA	NA	NA	NA
Yes	NA	NA	NA	NA
Low‐level viraemia (LLV)				
No LLV	(reference)		NA	NA
LLV	1.63 (1.38, 1.91)	<0.001	NA	NA
Viral load (copies/ml)				
≤50	NA	NA	(reference)	
51–199	NA	NA	1.39 (1.13, 1.69)	0.001
200–399	NA	NA	1.69 (1.33, 2.16)	<0.001
400–999	NA	NA	2.03 (1.63, 2.53)	<0.001

*Note*: Values are in frequency and % unless otherwise specified.

Abbreviations: 3TC, lamivudine; ABC, abacavir; ART, antiretroviral therapy; CI, confidence interval; DTG, dolutegravir; HR, hazard ratio; LLV, low‐level viraemia; LPV/r, lopinavir/ritonavir; NA, not applicable; TDF, tenofovir disoproxil fumarate; WHO, World Health Organization.

^a^Model 1 included the categorical ART regimen and dichotomous LLV variables.

^b^Model 2 included the categorical ART regimen and categorical LLV variables.

When defining VF as 200 or greater copies/ml, older age, WHO Stages II−IV, CD4 counts <500 cells/mm^3^, LLV and patients currently on second‐line ART were more likely to be associated with VL ≥200 copies/ml in univariable analysis (Table 4). Multivariable Cox regression analysis again demonstrated that age <5 years (aHR: 1.66; 95% CI, 1.03−2.68; *p* = 0.04) and immunosuppression (CD4 200–499 cells/mm^3^ [aHR: 1.64; 95% CI, 1.31−2.05; *p*<0.001] or <200 cells/mm^3^ [aHR: 1.99; 95% CI, 1.36−2.90; *p*<0.001]) were associated with VF (Table 5 and Table S2). Once more, DTG was found to be protective (aHR: 0.64; 95% CI, 0.54−0.75; *p*<0.001). LLV was associated with VF at all levels studied: overall (aHR: 3.85; 95% CI, 3.33−4.46; *p*<0.001), 51–199 copies/ml (aHR: 1.41; 95% CI, 1.15−1.72; *p* = 0.001), 200–399 copies/ml (aHR: 7.99; 95% CI, 6.68−9.57; *p*<0.001) and 400–999 copies/ml (aHR: 9.37; 95% CI, 7.85−11.18; *p*<0.001).

**Table 4:** Characteristics associated with virologic failure (viral load ≥200 copies/ml), univariable Cox proportional hazards regression

	Viral load <200	Viral load ≥200	Univariable	
Variables	(*n* = 1688)	(*n* = 930)	HR (95% CI)	*p*‐value
Age (years), median (IQR)	12.6 (9.1, 16.5)	14.1 (10.5, 17.1)	1.01 (1.00, 1.03)	0.08
Age group (years)				
<5	118 (7.0)	19 (2.0)	1.18 (0.73, 1.89)	0.51
5–9	387 (22.9)	174 (18.7)	(reference)	
10–14	572 (33.9)	333 (35.8)	0.87 (0.72, 1.04)	0.13
15–19	611 (36.2)	404 (43.4)	1.07 (0.90, 1.28)	0.44
Age at ART start, median (IQR)	3.9 (1.5, 7.8)	4.6 (1.7, 8.0)	1.03 (1.02, 1.05)	<0.001
Sex				
Female	870 (51.5)	506 (54.4)	(reference)	
Male	818 (48.5)	424 (45.6)	0.97 (0.85, 1.11)	0.66
Base WHO Stage				
I	466 (27.6)	191 (20.5)	(reference)	
II	313 (18.5)	179 (19.2)	1.11 (0.90, 1.36)	0.34
III	410 (24.3)	253 (27.2)	1.09 (0.90, 1.32)	0.36
IV	499 (29.6)	307 (33.0)	1.08 (0.90, 1.30)	0.4
Current WHO Stage				
I	160 (9.5)	31 (3.3)	(reference)	
II	158 (9.4)	56 (6.0)	1.31 (0.84, 2.02)	0.23
III	440 (26.1)	247 (26.6)	1.70 (1.17, 2.47)	0.01
IV	930 (55.1)	596 (64.1)	1.72 (1.20, 2.47)	0.003
Current weight (kg), median (IQR)	32.0 (22.4, 45.2)	36.0 (25.8, 49.0)	1.00 (1.00, 1.01)	0.57
Recent CD4 results (cells/mm^3^), median (IQR)	993.0 (716.0, 1328.0)	866.5 (611.5, 1190.0)	1.00 (1.00, 1.00)	<0.001
Recent CD4 results (cells/mm^3^)				
≥500	1543 (91.4)	808 (86.9)	(reference)	
200–499	113 (6.7)	93 (10.0)	1.92 (1.55, 2.38)	<0.001
<200	32 (1.9)	29 (3.1)	2.21 (1.52, 3.20)	<0.001
Time from ART start to first VL				
6–12 months	536 (31.8)	228 (24.5)	(reference)	
1–5 years	821 (48.6)	514 (55.3)	0.82 (0.70, 0.96)	0.01
>5 years	331 (19.6)	188 (20.2)	0.76 (0.63, 0.92)	0.01
Time from ART start to last VL, median (IQR)	6.5 (3.4, 9.2)	7.8 (5.8, 9.9)	0.97 (0.95, 0.99)	0.002
Tuberculosis				
No	1411 (83.6)	798 (85.8)	(reference)	
Yes	277 (16.4)	132 (14.2)	0.97 (0.81, 1.17)	0.78
Baseline ART regimens				
ABC+3TC+LPV/r	446 (26.5)	168 (18.1)	(reference)	
AZT+3TC+NV	403 (23.9)	253 (27.3)	0.78 (0.64, 0.95)	0.01
AZT+3TC+EFV	283 (16.8)	220 (23.7)	0.87 (0.71, 1.07)	0.2
ABC+3TC+EFV	169 (10.0)	117 (12.6)	0.98 (0.77, 1.24)	0.87
TDF+3TC+DTG	102 (6.1)	22 (2.4)	1.16 (0.75, 1.82)	0.5
d4T+3TC+NVP	128 (7.6)	72 (7.8)	0.76 (0.58, 1.00)	0.053
ABC+3TC+DTG	138 (8.2)	64 (6.9)	0.94 (0.70, 1.25)	0.66
Other	16 (0.9)	11 (1.2)	1.32 (0.72, 2.44)	0.37
Current ART regimens				
TDF+3TC+DTG	908 (53.8)	542 (58.3)	(reference)	
ABC+3TC+DTG	504 (29.9)	190 (20.4)	0.99 (0.84, 1.16)	0.87
ABC+3TC+LPV/r	116 (6.9)	49 (5.3)	1.75 (1.30, 2.34)	<0.001
Other	160 (9.5)	149 (16.0)	1.86 (1.55, 2.23)	<0.001
Current ART regimens				
First line	1528 (90.5)	781 (84.0)	(reference)	
Second line	160 (9.5)	149 (16.0)	1.81 (1.52, 2.16)	<0.001
Any DTG in current ART regimens				
No	259 (15.3)	183 (19.7)	(reference)	
Yes	1429 (84.7)	747 (80.3)	0.55 (0.46, 0.64)	<0.001
Any DTG in current second‐line ART regimens				
No	1678 (99.4)	921 (99.0)	(reference)	
Yes	10 (0.6)	9 (1.0)	1.55 (0.81, 3.00)	0.19
Low‐level viraemia (LLV)				
No LLV	1301 (77.1)	257 (27.6)	(reference)	
LLV	387 (22.9)	673 (72.4)	3.91 (3.38, 4.51)	<0.001
Viral load (copies/ml)				
≤50	1301 (77.1)	257 (27.6)	(reference)	
51–199	387 (22.9)	155 (16.7)	1.41 (1.15, 1.72)	0.001
200–399	0 (0.0)	241 (25.9)	7.82 (6.55, 9.34)	<0.001
400–999	0 (0.0)	277 (29.8)	9.11 (7.67, 10.82)	<0.001

*Note*: Values are in frequency and % unless otherwise specified. Differences in participant characteristics between groups (viral load ≥200 vs. viral load <200) were determined by the chi‐square or Fisher's exact tests for categorical variables and the Kruskal−Wallis test for continuous variables.

Abbreviations: 3TC, lamivudine; ABC, abacavir; ART, antiretroviral therapy; AZT, zidovudine; d4T, stavudine; DTG, dolutegravir; EFV, efavirenz; IQR, interquartile range; LLV, low‐level viraemia; LPV/r, lopinavir/ritonavir; NVP, nevirapine; TDF, tenofovir disoproxil fumarate; VL, viral load; WHO, World Health Organization.

**Table 5:** Characteristics associated with virologic failure (viral load ≥200 copies/ml), multivariable Cox proportional hazards regression

	Multivariable, model 1^a^		Multivariable, model 2^b^	
Variables	HR (95% CI)	*p*‐value	HR (95% CI)	*p*‐value
Age group (years)				
<5	1.66 (1.03, 2.68)	0.04	1.93 (1.19, 3.13)	0.01
5–9	(reference)		(reference)	
10–14	0.87 (0.70, 1.07)	0.19	0.84 (0.68, 1.04)	0.11
15–19	1.07 (0.85, 1.35)	0.58	1.00 (0.79, 1.27)	0.99
Sex				
Female	(reference)		(reference)	
Male	0.97 (0.85, 1.10)	0.63	1.01 (0.88, 1.15)	0.92
Current WHO Stage				
I	(reference)		(reference)	
II	1.13 (0.73, 1.77)	0.58	1.23 (0.79, 1.93)	0.35
III	1.19 (0.81, 1.75)	0.37	1.09 (0.75, 1.61)	0.64
IV	1.20 (0.83, 1.74)	0.33	1.05 (0.72, 1.52)	0.8
Recent CD4 results (cells/mm^3^)				
≥500	(reference)		(reference)	
200–499	1.64 (1.31, 2.05)	<0.001	1.59 (1.28, 1.99)	<0.001
<200	1.99 (1.36, 2.90)	<0.001	1.86 (1.27, 2.71)	0.001
Tuberculosis				
No	(reference)		(reference)	
Yes	1.00 (0.83, 1.20)	0.99	1.14 (0.94, 1.38)	0.18
Current ART regimens				
TDF+3TC+DTG	(reference)		(reference)	
ABC+3TC+DTG	1.17 (0.94, 1.45)	0.16	1.21 (0.97, 1.50)	0.09
ABC+3TC+LPV/r	1.80 (1.31, 2.45)	<0.001	1.89 (1.38, 2.60)	<0.001
Other	1.64 (1.37, 1.98)	<0.001	1.70 (1.41, 2.05)	<0.001
Any DTG in current ART regimens				
No	NA	NA	NA	NA
Yes	NA	NA	NA	NA
Low‐level viraemia (LLV)				
No LLV	(reference)		NA	NA
LLV	3.85 (3.33, 4.46)	<0.001	NA	NA
Viral load (copies/ml)				
≤50	NA	NA	(reference)	
51–199	NA	NA	1.41 (1.15, 1.72)	0.001
200–399	NA	NA	7.99 (6.68, 9.57)	<0.001
400–999	NA	NA	9.37 (7.85, 11.18)	<0.001

*Note*: Values are in frequency and % unless otherwise specified.

Abbreviations: 3TC, lamivudine; ABC, abacavir; ART, antiretroviral therapy; CI, confidence interval; DTG, dolutegravir; HR, hazard ratio; LLV, low‐level viraemia; LPV/r, lopinavir/ritonavir; NA, not applicable; TDF, tenofovir disoproxil fumarate; WHO, World Health Organization.

^a^Model 1 included the categorical ART regimen and dichotomous LLV variables.

^b^Model 2 included the categorical ART regimen and categorical LLV variables.

**Figure 3:**
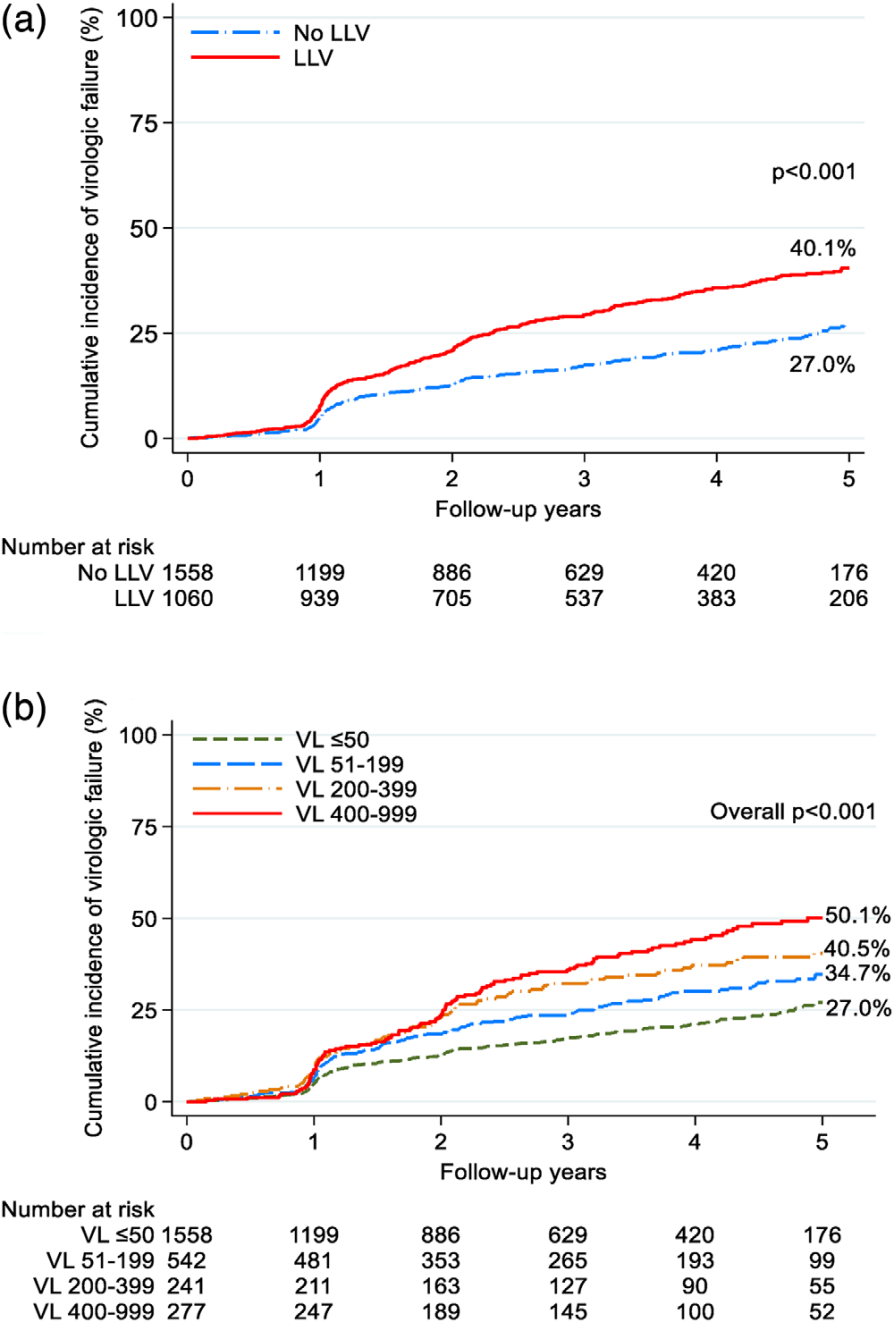
(A) Cumulative incidence of virologic failure, by LLV. (B) Cumulative incidence of virologic failure, by LLV category. *p*‐value was obtained from the log‐rank test. LLV, low‐level viraemia; VL, viral load.

**Figure 4:**
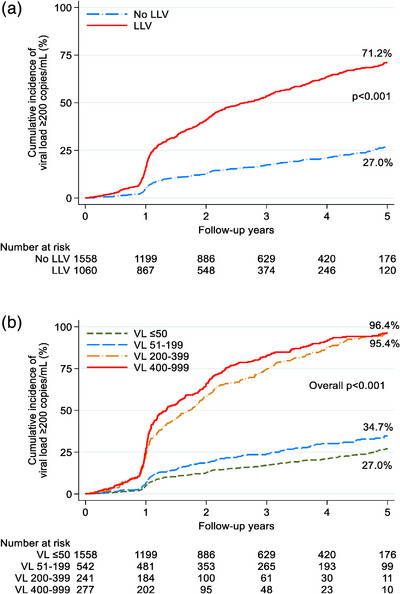
(A) Cumulative incidence of viral load ≥200 copies/ml, by LLV. (B) Cumulative incidence of viral load ≥200 copies/ml, by LLV category. *p*‐value was obtained from the log‐rank test. LLV, low‐level viraemia; VL, viral load.

Kaplan−Meier estimates of VF at VL ≥1000 copies/ml showed cumulative incidence of 40.1% for any LLV, 34.7% for VL 50–199 copies/ml, 40.5% for VL 200–399 copies/ml and 50.1% for 400–999 copies/ml, compared to 27.0% for no LLV (*p*<0.001) after 5 years of follow‐up (Figure 3A,B). When VF was defined as VL ≥200 copies/ml, cumulative incidence rose to 71.2% for any LLV versus 27.0% for no LLV (*p*<0.001) and 34.7% for VL 50–199 copies/ml, 95.4% for VL 200–399 copies/ml and 96.4% for 400–999 copies/ml (*p* < 0.001) (Figure 4A,B).

## 4 DISCUSSION

In this multicentre retrospective cohort study, we demonstrated that LLV in CALHIV on ART was a risk factor for developing VF at Baylor Foundation Tanzania sites in Mbeya and Mwanza. The association between LLV and VF was found when defining VF as either ≥1000 or ≥200 copies/ml. Moreover, when stratifying LLV into VL levels, higher levels of LLV were found to be associated with a higher risk of VF.

In this large cohort of 2618 children, LLV was quite common, affecting 40% of the participants after their initial baseline undetectable VL. This number is high compared to the LLV rate of 17% in a study among Asian children [30], but similar to the 42% found in a small study among youth in the United States [31]. In a study among South African adults, LLV was found in 23% on first‐line ART and in 26% of participants on second‐line ART [16] (our study showed 40% LLV for first‐line ART and 47% for second‐line ART).

Our study demonstrated an association between LLV and VF, whether VF was defined as a VL of ≥1000 or ≥200 copies/ml. This finding aligns with previous studies that have shown a similar correlation between LLV and VF, although most of these studies focused on adult populations. A similar study in children found that having at least one episode of LLV after viral suppression conferred a risk of VF, defined as VL ≥1000 copies/ml, with an aHR of 3.01 [30]. When compiling studies involving adults, aHRs have ranged from 2.2 to 4.1 [13–16, 32] for any level of LLV. A large study among adults in South Africa demonstrated a higher risk of VF with higher levels of LLV [16]. Interestingly, studies are not consistent in showing the risk of VF at lower levels of LLV, especially at LLV <200 copies/ml [33], as was observed in our study.

One difficulty in comparing studies is the variety of definitions of VF or LLV. In our study, we only looked for LLV after a VL measurement of <50 copies/ml in an attempt to reduce bias created by external factors, for example poor caregiver support. Moreover, in contrast to current WHO guidelines defining VF as two VL measurements ≥1000 copies/ml [34], we chose to define VF as a single unsuppressed VL. This decision was made due to the substantial increase in clinical services for CALHIV with VF, which is common in LMICs. For our Viremia Clinic, CALHIV with VF receive individualized care tailored to their specific challenges and includes counselling, social support, peer support and other services.

As mentioned previously, current studies using assays that can detect DRMs at low level of the virus have found that people living with HIV with LLV have a significant amount of antiretroviral resistance [18, 20, 35]. This is especially salient in the current era of ART where many countries are depending on the durability of DTG for success in their HIV programmes. The best way to ensure the extended use of DTG is to prioritize the prevention of DRMs and limit their spread once they do arise.

One current issue has been the uncertainty over whether drug selection, especially the addition of DTG, has an influence on the level of LLV experienced and whether DRMs are still a risk with LLV while on a DTG [34]. Most studies of LLV were performed before the widespread use of DTG. An African Cohort study (AFRICOS) in African adults found that PI use was associated with LLV [33], a finding which we did not replicate; however, the comparison is difficult to make because, as noted previously, paediatric formulations of PIs are often unpalatable and poorly tolerated. Interestingly, in our study, we found that being on second‐line ART was associated with LLV (*p* = 0.01), while DTG use was not protective against LLV (*p* = 0.39). We observed that over 80% of the participants were on a DTG‐containing regimen and as seen in Figure 4, LLV has been steadily increasing over time (in conjunction with the increase in DTG use since 2019). While we are not able to comment on DRMs in this cohort, we did observe a high level of VF that has persisted despite the introduction of DTG.

The WHO recently reinforced its recommendation of ≥1000 copies/ml for defining VF in LMICs [12]. They referenced a recent study demonstrating that while the standard “Undetectable = Untransmittable (U = U)” cutoff of 200 copies/ml is essentially protective for sexual transmission of HIV [36], very few transmission events occur between 200 and 1000 copies/ml [37]. However, if LLV predisposes one to future VF, then LLV should be viewed as a step on the path to VF. This perspective is especially important in LMICs where VL is only taken sporadically (our study had an average of 1.0 VL per year). Moreover, many paediatric HIV programmes are treating increasing numbers of adolescents where sexual transmission of HIV must be considered. Therefore, ignoring LLV likely puts CALHIV at risk for poor treatment outcomes and increased sexual transmission.

Multiple groups have advocated for changing VF guidelines [16, 33, 35]; however, there is some variation on what VL value should be considered “suppressed.” Another challenge that arises due to the relatively low number of VLs performed per year in LMICs is identifying viral blips. Our analysis shows that if VF is defined as ≥200 copies/ml, there is a large increase in the risk of VF between LLV of 50−200 copies/ml (aHR: 1.41; 95% CI, 1.15−1.72) versus LLV of 200−399 copies/ml (aHR: 7.99; 95% CI, 6.68−9.57). Therefore, we suggest changing global guidelines to define VF as a VL greater than 200 copies/ml.

This study had several limitations. Due to its retrospective design, several patient characteristics were not able to be addressed, for example adherence or socio‐economic factors. Moreover, our data was missing certain elements of patient history including ART taken before clinic enrolment or prevention of vertical transmission services that may have been received at birth. Additionally, while genotyping is available at our clinics, there are significant barriers and delays that result in few CALHIV having DRM information available.

Lastly, our study did not consider the length of time that participants experienced LLV. We took the highest level of LLV in between an undetectable VL and the last recorded value. Due to the retrospective nature of the analysis, we were limited by national guidelines dictating that CALHIV only have one VL measurement per year, unless they experience a VL ≥1000 copies/ml. The low frequency in VLs makes it difficult to determine whether LLV is isolated or persistent. However, we believe our findings are clinically relevant and reflect the challenges most clinicians in LMICs are facing.

## 5 CONCLUSIONS

LLV is associated with increased risk for VF in CALHIV. The risk of VF increases with higher levels of LLV. LLV should be viewed as early VF and thus should be managed with urgency utilizing interventions such as peer support, adherence counselling and timely repeat VLs. Neglecting LLV can lead to the development and spread of DRMs and can result in poor treatment outcomes in CALHIV, especially those living in LMICs.

## COMPETING INTERESTS

The authors declare that they have no competing interests.

## AUTHORS’ CONTRIBUTIONS

KPM conceived and designed the study. KPM and DTN accessed and verified the underlying data. DTN cleaned the data and performed the analysis. LBK, EWK, NEK and ENM coordinated data collection. LFM supervised and administered the project. KPM wrote the first draft. All authors participated in writing the final draft and approved the final version.

## FUNDING

No funding was received for this project.

## ETHICS APPROVAL STATEMENT

Ethical approval for this activity was granted by the Baylor College of Medicine Institutional Review Board for retrospective medical audits (H32491) performed at the Baylor College of Medicine Children's Foundation—Tanzania sites in Mbeya and Mwanza, Tanzania.

## Supporting information




**Table S1**. Characteristics associated with virological failure (VL ≥1000 copies/mL), multivariable Cox proportional hazards regression.
**Table S2**. Characteristics associated with virologic failure (viral load ≥200 copies/mL), multivariable Cox proportional hazards regression.

## Data Availability

The data that support the findings of this study are available on request from the corresponding author. The data are not publicly available due to privacy or ethical restrictions.
