# Supplementary material for: Low‐level viraemia as a risk factor for virologic failure in children and adolescents living with HIV on antiretroviral therapy in Tanzania: a multicentre, retrospective cohort study
Source: J Int AIDS Soc. 2025 May 12;28(5):e26474. doi: 10.1002/jia2.26474 (PMC12069799; doi:10.1002/jia2.26474)
Supplement: Supplementary file 1 — Table S1. Characteristics associated with virological failure (VL ≥1000 copies/mL), multivariable Cox proportional hazards regression. Table S2. Characteristics associated with virologic failure (viral load ≥200 copies/mL), multivariable Cox proportional hazards regression. [file JIA2-28-e26474-s001.docx]

**Table S1. Characteristics associated with virological failure (VL ≥1000 copies/mL), multivariable Cox proportional hazards regression**

| Variables | Multivariable, model 3‡ | | Multivariable, model 4§ | |
| --- | --- | --- | --- | --- |
|  | **HR (95% CI)** | **p-value** | **HR (95% CI)** | **p-value** |
| Age group (years) |  |  |  |  |
| <5 | 2.18 (1.26, 3.77) | 0.01 | 2.23 (1.29, 3.87) | 0.004 |
| 5-9 | (reference) |  | (reference) |  |
| 10-14 | 0.99 (0.78, 1.25) | 0.92 | 1.00 (0.79, 1.26) | 1.00 |
| 15-19 | 1.17 (0.93, 1.48) | 0.18 | 1.17 (0.93, 1.48) | 0.18 |
| Sex |  |  |  |  |
| Female | (reference) |  | (reference) |  |
| Male | 0.93 (0.79, 1.09) | 0.37 | 0.93 (0.79, 1.09) | 0.37 |
| Current WHO stage |  |  |  |  |
| I |  |  | (reference) |  |
| II | 1.26 (0.72, 2.20) | 0.41 | 1.28 (0.74, 2.24) | 0.38 |
| III | 1.28 (0.79, 2.09) | 0.31 | 1.26 (0.78, 2.05) | 0.35 |
| IV | 1.45 (0.90, 2.31) | 0.12 | 1.44 (0.90, 2.31) | 0.13 |
| Recent CD4 results (cells/mm3) |  |  |  |  |
| ≥500 | (reference) |  | (reference) |  |
| 200-499 | 1.75 (1.35, 2.26) | <0.001 | 1.72 (1.33, 2.22) | <0.001 |
| <200 | 2.59 (1.73, 3.88) | <0.001 | 2.58 (1.72, 3.86) | <0.001 |
| Tuberculosis |  |  |  |  |
| No | (reference) |  | (reference) |  |
| Yes | 1.10 (0.89, 1.38) | 0.38 | 1.13 (0.91, 1.41) | 0.27 |
| Current ART regimens |  |  |  |  |
| TDF+3TC+DTG | NA | NA | NA | NA |
| ABC+3TC+DTG | NA | NA | NA | NA |
| ABC+3TC+LPV/r | NA | NA | NA | NA |
| Other | NA | NA | NA | NA |
| Any DTG in current ART regimens |  |  |  |  |
| No | (reference) |  | (reference) |  |
| Yes | 0.55 (0.45, 0.67) | <0.001 | 0.55 (0.45, 0.67) | <0.001 |
| Low-level viremia (LLV) |  |  |  |  |
| No LLV | (reference) |  | NA | NA |
| LLV | 1.63 (1.38, 1.91) | <0.001 | NA | NA |
| Viral load (copies/mL) |  |  |  |  |
| ≤50 | NA | NA | (reference) |  |
| 51-199 | NA | NA | 1.39 (1.13, 1.69) | 0.001 |
| 200-399 | NA | NA | 1.69 (1.33, 2.15) | <0.001 |
| 400-999 | NA | NA | 2.03 (1.63, 2.53) | <0.001 |

Values are in frequency and % unless otherwise specified. 3TC, lamivudine; ABC, abacavir; ART, antiretroviral therapy; CI, confidence interval; DTG, dolutegravir; HR, hazard ratio; LLV, low-level viremia; LPV/r, lopinavir/ritonavir; NA, not applicable; TDF, tenofovir disoproxil fumarate; WHO, World Health Organization. **‡**Model 3 included the dichotomous ART regimen and dichotomous LLV variables. **§**Model 4 included the dichotomous ART regimen and categorical LLV variables.

**Table S2. Characteristics associated with virologic failure (viral load ≥200 copies/mL), multivariable Cox proportional hazards regression**

| Variables | Multivariable, model 3‡ | | Multivariable, model 4§ | |
| --- | --- | --- | --- | --- |
|  | **HR (95% CI)** | **p-value** | **HR (95% CI)** | **p-value** |
| Age group (years) |  |  |  |  |
| <5 | 1.70 (1.05, 2.74) | 0.03 | 1.99 (1.23, 3.22) | 0.01 |
| 5-9 | (reference) |  | (reference) |  |
| 10-14 | 0.81 (0.67, 0.98) | 0.03 | 0.77 (0.64, 0.93) | 0.01 |
| 15-19 | 0.96 (0.80, 1.16) | 0.69 | 0.89 (0.74, 1.06) | 0.19 |
| Sex |  |  |  |  |
| Female | (reference) |  | (reference) |  |
| Male | 0.97 (0.85, 1.11) | 0.67 | 1.01 (0.89, 1.15) | 0.85 |
| Current WHO stage |  |  |  |  |
| I | (reference) |  | (reference) |  |
| II | 1.13 (0.72, 1.76) | 0.59 | 1.23 (0.79, 1.92) | 0.36 |
| III | 1.18 (0.80, 1.73) | 0.4 | 1.08 (0.74, 1.59) | 0.69 |
| IV | 1.20 (0.83, 1.73) | 0.34 | 1.05 (0.72, 1.52) | 0.81 |
| Recent CD4 results (cells/mm3) |  |  |  |  |
| ≥500 | (reference) |  | (reference) |  |
| 200-499 | 1.64 (1.31, 2.05) | <0.001 | 1.59 (1.27, 1.98) | <0.001 |
| <200 | 1.98 (1.36, 2.89) | <0.001 | 1.84 (1.26, 2.69) | 0.002 |
| Tuberculosis |  |  |  |  |
| No | (reference) |  | (reference) |  |
| Yes | 1.00 (0.83, 1.21) | 0.99 | 1.15 (0.95, 1.39) | 0.14 |
| Current ART regimens |  |  |  |  |
| TDF+3TC+DTG | NA | NA | NA | NA |
| ABC+3TC+DTG | NA | NA | NA | NA |
| ABC+3TC+LPV/r | NA | NA | NA | NA |
| Other | NA | NA | NA | NA |
| Any DTG in current ART regimens |  |  |  |  |
| No | (reference) |  | (reference) |  |
| Yes | 0.64 (0.54, 0.75) | <0.001 | 0.62 (0.52, 0.73) | <0.001 |
| Low-level viremia (LLV) |  |  |  |  |
| No LLV | (reference) |  | NA | NA |
| LLV | 3.82 (3.31, 4.42) | <0.001 | NA | NA |
| Viral load (copies/mL) |  |  |  |  |
| ≤50 | NA | NA | (reference) |  |
| 51-199 | NA | NA | 1.40 (1.14, 1.71) | 0.001 |
| 200-399 | NA | NA | 7.95 (6.64, 9.52) | <0.001 |
| 400-999 | NA | NA | 9.24 (7.74, 11.02) | <0.001 |

Values are in frequency and % unless otherwise specified. 3TC, lamivudine; ABC, abacavir; ART, antiretroviral therapy; CI, confidence interval; DTG, dolutegravir; HR, hazard ratio; LLV, low-level viremia; LPV/r, lopinavir/ritonavir; NA, not applicable; TDF, tenofovir disoproxil fumarate; WHO, World Health Organization. **‡**Model 3 included the dichotomous ART regimen and dichotomous LLV variables. **§**Model 4 included the dichotomous ART regimen and categorical LLV variables.
